# Contraception after in vitro fertilisation (IVF): a qualitative study of the views of women who have had spontaneous pregnancies after successful IVF

**DOI:** 10.1186/s12978-022-01349-2

**Published:** 2022-02-08

**Authors:** Annette Thwaites, Jennifer Hall, Geraldine Barrett, Judith Stephenson

**Affiliations:** EGA Institute of Women’s Health, University College London, Medical School Building, 74 Huntley Street, London, WC1E 6AU UK

**Keywords:** Contraception, Fertilization in vitro, Infertility, Qualitative

## Abstract

**Background:**

The use of in vitro fertilisation (IVF) has increased rapidly since its inception in 1978. Women seeking IVF have a wide range of subfertility causes including unexplained subfertility. A growing subgroup of women seek treatment for other reasons than fertility problems, for example, women in same sex relationships and single women. This study aims to better understand the contraceptive needs of women after successful IVF pregnancy in order to improve service delivery and prevent unplanned and rapid-repeat pregnancies.

**Methods:**

A qualitative study of views of women who have had spontaneous pregnancies after successful IVF. Participants were recruited using purposive and snowballing sampling methods from social media and peer networks. The framework method was used for analysis using NVivo12.

**Results:**

The sample comprised 21 interviewees from the United Kingdom (UK), having a range of spontaneous pregnancy outcomes, including single and multiple livebirths, miscarriage, ectopic pregnancy and termination of pregnancy. Contraceptive choices were subject to a complex and dynamic interaction of influencing factors including beliefs regarding subfertility, desire for children and views on contraception. None of the women recalled receiving any information or useful counselling about contraception during fertility or maternity care. After IVF pregnancy, most women (n = 16) used no or ineffective contraception. Spontaneous pregnancy was not universally welcomed in this group and inter-pregnancy intervals were often short (n = 16, less than 18 months). Even after subsequent spontaneous pregnancy, use of contraception and the most effective methods remained low. Women held persistent beliefs regarding their subfertility despite subsequent spontaneous pregnancy. They associated aspects of the IVF process with a sense of personal failure, despite an ultimately “successful” outcome of livebirth. These aspects may reinforce their self-belief in subfertility. Other barriers to contraception use in women having IVF included: lack of knowledge of likelihood of spontaneous pregnancy, lack of contraceptive experience and inherent incentives towards shorter inter-pregnancy intervals.

**Conclusions:**

The contraceptive needs of women having IVF pregnancies are real and are being overlooked. Fertility services should take responsibility for providing information on the risks of subsequent spontaneous pregnancy. Maternity and community healthcare professionals must address women’s perceptions of their fertility in order to engage them in contraception counselling.

## Plain English summary

Women who have difficulty getting pregnant may choose to use fertility treatment to help. Fertility treatment known as ‘in vitro fertilisation’, or IVF, was developed around 40 years ago and has been increasingly used ever since. Over 8 million babies have been born this way around the world. There are many different reasons why women can have problems getting pregnant and in a quarter of cases the cause is never found. We know that some women, who have babies using IVF, get pregnant again afterwards without fertility treatment. Getting pregnant again quickly, or when a woman isn’t ready, can be worse for the health of the mother and child. Therefore, this study aims to understand what contraception women need after IVF to plan and space their future pregnancies. We spoke to 21 women who became pregnant without fertility treatment after having their first babies using IVF. Although most of these women had not been using contraception after their first baby, they were shocked to get pregnant again as they firmly believed they could not get pregnant without fertility treatment. Most of these pregnancies had happened quickly and not all the women were happy to be pregnant again. These women were not given the information they needed about their chances of getting pregnant after IVF without fertility treatment. Fertility and maternity services must close this gap. Health professionals also need to explore women’s beliefs about their own fertility after IVF before they can start to have helpful conversations about contraception.

## Background

In a climate of increasing age of conception and availability of assisted reproductive technology (ART), the number of women seeking assisted conception has grown rapidly [1]. More than eight million babies have been born via in vitro fertilisation (IVF) since the first in 1978 [2] and 1–5% of babies born in the developed world today are conceived via IVF [3]. In the UK the number of IVF cycles per year has more than tripled since 1994 (18,304 cycles) to 2018 (68,724 cycles) [1]. It is estimated that one in seven women has difficulty conceiving. This is a heterogeneous group with a wide range of underlying male and female causative factors and, importantly, in 25% of couples seeking treatment, no identifiable cause is found [4]. Developments in ART, together with wider social change, have led to increasingly diverse reasons for the use of fertility treatment. According to Human Fertilisation and Embryology Authority (HFEA) data, in the UK in 2018, 94.4% of all treatment cycles were undertaken by women in heterosexual relationships, 3.1% by women in same sex relationships, 2% by single women and 0.5% by surrogates [1]. Whilst heterosexual partnerships are still clearly the majority group, this is also the group that is growing most slowly. In addition, 1% of assisted conception cycles are now for couples seeking to screen for serious genetic conditions using preimplantation genetic diagnosis (PGD) and the number of conditions that can be detected by these methods is increasing. None of these growing subgroups of women are accessing fertility treatments primarily for fertility problems.

The evidence relating to spontaneous conception after assisted conception varies widely according to population, cause of subfertility, type and outcome of fertility treatment and length of follow-up. A recent large retrospective UK cohort study estimated the treatment-independent livebirth rate after IVF livebirth over 5 year follow up as fifteen per hundred women (or one in seven) which peaks in the first few years after fertility treatment [5]. This study did not include consideration of contraception nor the plannedness of the subsequent pregnancy. Surprisingly little is known about the circumstances or impact of spontaneous pregnancy after IVF. Recent media articles present a spectrum of views and outcomes from “welcomed blessing” [6] to abortion [7]. To our knowledge this is the first study focussed on the use and need for contraception of women who have had IVF in the UK. The aim is to better understand the contraceptive needs of women after IVF pregnancy in order to improve delivery of services and prevent unplanned and rapid-repeat pregnancies in this group.

## Methods

Given the scarcity of evidence on spontaneous pregnancy after IVF, an inductive qualitative approach was chosen to explore individual experiences, comparing detailed observations to generate an understanding of underlying contraceptive need.

The specific objectives were:

(i) To determine how reproductive experiences of subfertility, fertility treatment, and subsequent spontaneous conception influence women’s perception of their own fertility and contraception choices;
(ii) To identify specific barriers and enablers to providing effective contraceptive counselling and immediate and ongoing postnatal contraception to women after IVF;
(iii) To develop recommendations as to how UK services can best help women to plan and space their future pregnancies optimally after IVF.

### Data collection

In-depth interviews were selected to enable rapport-building and facilitate sharing of sensitive and personal topics. One-off, hour-long interviews were conducted by AT in September/October 2020. Online interviewing, via Zoom and Microsoft Teams, was undertaken to widen the potential geographical location of participants and accommodate practical budgetary, time constraints and additional restrictions related to COVID-19. The interviews often took place in the evenings due to occupational and childcare commitments. The topic guide included questions on pregnancy preferences, planning, spacing and outcomes, contraception use and fertility treatment. Similar broad, open questions were used to introduce each topic and subsequent probes varied according to the individual’s narrative style and experiences. The interviews were audio recorded and transcribed verbatim by a professional transcription service. Electronic field notes of contextual observations and initial reflections were made immediately after the interview. The recordings, transcripts and field notes were securely stored in the UCL Data Safe Haven (DSH).

### Sampling and recruitment

The following inclusion criteria were selected:

- Women aged over 18
- who have had a livebirth conceived via IVF in the UK
- who have had a subsequent pregnancy without fertility treatment
- able to read and speak English

The sample, of women who have had spontaneous pregnancies after IVF, was selected as these women have proven fertility. They therefore potentially have ongoing or future need for contraception, may have had an unmet need for contraception, and may also have experienced a shift in their perception of fertility and/or contraceptive need.

Participants were identified via a combination of purposive and snowballing methods and recruited from a range of sources including social media and peer networks. Women were included who had a range of different spontaneous pregnancy outcomes (after IVF livebirths), including livebirth, miscarriage and termination of pregnancy (TOP). We also sought to include women who were currently pregnant with a spontaneous pregnancy who may have unique insights or recall certain aspects of their reproductive journey more readily. The sample was also chosen to include women from a spread of geographical regions across the UK.

The desired sample size was approximately 20 women. Approximately 20,000 livebirths in the UK result from IVF per year [1] and some studies estimate that approximately 3000 of those women may go on to have spontaneous pregnancies in following 5 years [5]. The chosen sample size therefore reflects a balance between the relatively small population of interest and the need to capture a range of views through the lens of individual, complex, reproductive life stories.

### Data analysis

A detailed thematic analysis was undertaken using the Framework Method [8]. This involved five stages of: familiarisation, identifying a thematic framework, indexing, charting, mapping and interpretation. Familiarisation was undertaken by listening to the interviews to identify key ideas and common themes. The transcriptions were imported into NVivo12 and a thematic framework developed which consisted of a hierarchical nodal structure from those preidentified in the topic guide together with emergent themes. The nodes included descriptive categories (e.g. postnatal contraception use) and analytical concepts (e.g. barriers to postnatal contraception). The data were then coded and the framework reviewed for intranodal consistency and internodal coherence and iterative changes made as necessary. The field notes were also used to integrate contextual factors. Charting was undertaken in Excel (across discrete variables) and NVivo. A framework matrix was constructed of perception of fertility, desire for a child and contraception use across three different time periods: pre IVF, post IVF and post subsequent spontaneous pregnancy. The final mapping and interpretation stages of analysis, involved writing and testing ideas and relationships between nodes and attributes to address the key objectives of the study and construct illustrative concept maps (Figs. 2 and 3).

## Results

### The sample

The sample contained 21 women, 26 IVF pregnancies and 28 spontaneous pregnancies.

The sample was relatively homogenous with respect to key demographic data. The women were aged between 35 and 50 years, with most being in their 40s (14/21). All spoke English as their first language and nearly all described themselves as White (20/21) or White British (18/21). All women except one (P12) were married and all were living in nuclear family households i.e. with their children and their husband or long-term male partner. All children except one (donor egg conception) were the biological children of the interviewee and her current husband/partner; the duration of these partnerships was 10 or more years. Women lived and had had IVF treatment across England and Scotland.

Women identified as Christian or Catholic (10/21), none/atheist/agnostic (9/21) and Jewish (2/21). However, most women spontaneously qualified their answer with an absence of religiosity e.g. “Christian, but I am not a massively practising one” (P20); “I wouldn’t say that it’s a huge part of my life at the moment” (P21). All the women except P9 denied that religion affected their contraceptive or reproductive choices. One woman was married to a priest and said religion had “quite a significant effect” on their fertility choices, influencing their decision not to talk openly about IVF treatment as “there are certain people in church who wouldn’t approve of IVF” (P9).

Twelve women were doctors, a further two were allied healthcare professionals (physiotherapist and health visitor) and one was employed in health services research. Three women had non-health related careers and three were not currently employed. The doctors’ specialisms included those with professional experience of contraception, pregnancy and/or fertility (general practice, obstetrics, paediatrics and sexual health). As expected from this occupational profile, there was high-level educational attainment across the sample. Five participants had or were pursuing PhDs and all women had degrees except one (P3) who left formal education aged 16. The women also had multiple, additional, professional qualifications, memberships, authorships and positions of responsibility. In response to the first open question ‘tell me about yourself’ all women spontaneously mentioned work perhaps reflecting the high proportion of professional identity and demanding careers. Fifteen women spontaneously mentioned how their career had impacted their family planning and reproductive timelines.

**Fig. 1:**
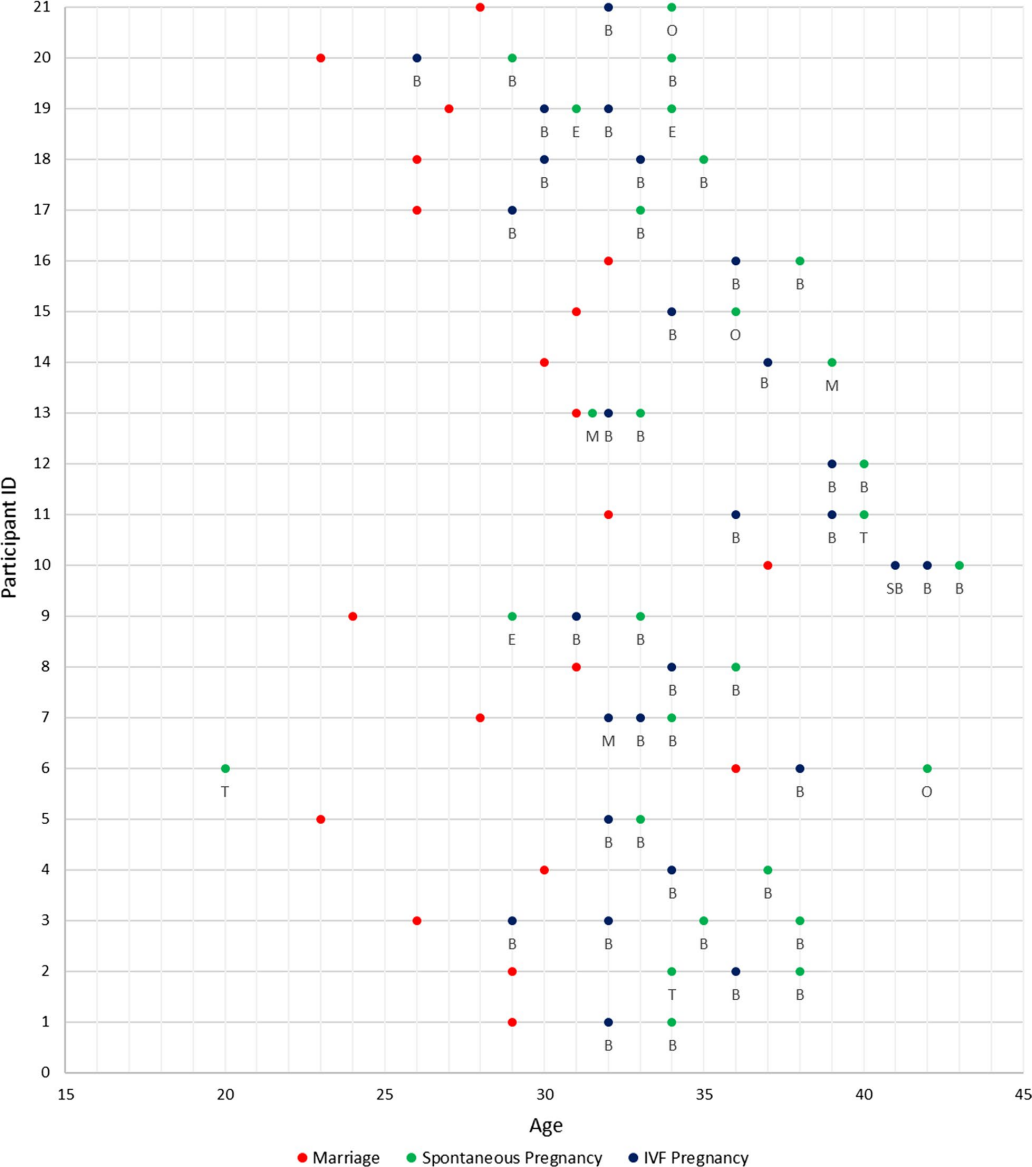
Pregnancies by age, type and outcome. Birth (B), Stillbirth (SB), Ectopic Pregnancy (E), Miscarriage (M), Termination of Pregnancy (T), Ongoing (O). P7 first pregnancy: Intrauterine Insemination. P19 second pregnancy: unknown whether spontaneous or result of a frozen IVF cycle

In contrast to the uniformity of demographic data, there is striking diversity across the sample in terms of reproductive outcomes, cause and duration of subfertility. Women had between one and five children, with most women (13/21) having two. The children of the sample included four sets of twins (three IVF and one spontaneous) (P3, P9, P14, and P13 respectively) and a spontaneous triplet pregnancy (ongoing at the time of interview as a twin pregnancy, P21). All women had their first child via IVF but four women had had a spontaneous pregnancy prior to their IVF pregnancies (with outcomes of miscarriage, ectopic and two terminations). Figure 1 summarises the sample pregnancies by age, type and outcome. The time since a woman’s last pregnancy varied from 0 to 11 years with three women pregnant at the time of interview (P6, P15, and P19) and three women experiencing their last pregnancy 10 or more years ago (P5, P7, P14). Most pregnancies occurred when women were aged in their 30s. Age at first pregnancy ranged between 20 and 41 years and age at first IVF pregnancy between 26 and 41 years with average age at first IVF of 32 years.

Spontaneous pregnancies after IVF included the full range of pregnancy outcomes: livebirth, multiple livebirths, ectopic pregnancy, miscarriage and termination of pregnancy. Two women had two sequential spontaneous pregnancies after their IVF pregnancies (P3 and P20). The sample contained women with unexplained, male factor, tubal, anovulatory and joint infertility (see Table 1). Time trying to conceive until first livebirth, as estimated from the interview responses, ranged between 1 and 9 years with the majority (12/21) conceiving within three years and four women taking more than five years.

**Table 1:** Cause and duration of subfertility

ID	Cause of subfertility	Time trying to conceive before 1st livebirth, years
1	Unexplained	3
2	PCOS, male factor	1.5
3	PCOS	3–5
4	Unexplained, borderline male factor	4
5	Endometriosis	9
6	Tubal factor	2
7	Unexplained, borderline male factor	5
8	PCOS	3
9	Complications from ectopic surgery	3
10	Ovarian insufficiency	6
11	Unexplained, borderline male & female factors	2
12	Low anti-müllerian hormone, male factor	3
13	PCOS, hypothyroid	1
14	Male factor	4
15	PCOS	4
16	Male factor	3
17	PCOS, borderline male factor	6
18	Tubal factor, unilateral laparoscopic tubal ligation, PCOS	2
19	Tubal factor	3
20	PCOS, male factor	4
21	Unexplained	1

### Reproductive and contraceptive summary

After IVF pregnancy resulting in livebirth, almost all the women (20/21) firmly believed that they were very unlikely to conceive naturally. These beliefs were based on factors including age, nature and duration of subfertility, failed assisted conception treatments and specific experiences of IVF. Only one woman (P8) commented that she had a cautious optimism regarding her chances of subsequent spontaneous conception, (associated with new onset of a regular menstrual cycle). Whilst the vast majority of women wanted more children (18/21) after their IVF livebirths, three women (P10, P11, P14) did not. Four women qualified their desires for further pregnancy after IVF birth, volunteering that they were *not* prepared to undertake further IVF. Six women specified that they did not want a child in the immediate or early postnatal period, and this corresponded well to postpartum contraception use. However, most women (n = 16) used no contraception (n = 11) or ineffective methods (inconsistent condom use or withdrawal) (n = 5) before their next pregnancy with three reporting consistent condom use and only two women using any hormonal method (progesterone-only pill).

There were 23 spontaneous pregnancies after IVF (16 to women with one child and 7 who already had two or more children). Whilst most women (n = 16) were happy to have conceived spontaneously, fourteen stated that this had occurred sooner than they had expected and eight of these women described wanting a longer gap before this conception.

The inter-pregnancy intervals were often short (n = 16, less than 18 months) or very short (n = 7, less than 12 months). Three women (P1, P11, P18) subsequently associated their rapid repeat spontaneous pregnancies with weaning. Five women in the sample (P5, P7, P8, P13, P18) expressed a preference for a short gap after the birth of their first child, all aged 32–34 at that time. Nine women had made a conscious decision to try and conceive naturally, as evidenced by stopping contraception, at the time of their spontaneous conceptions. A further four women had initiated or planned further IVF at the time of their spontaneous conception. Two of these women (P15, P21) were waiting for IVF clinics to reopen during the exceptional, extended closure due to COVID-19 [9]. Five women had mixed or negative feelings towards the pregnancy, four actively considered termination and one woman opted for a termination of pregnancy. In addition, partners’ desires for more children were not necessarily aligned with those of the woman at the time of spontaneous conception. Other impacts of unplanned spontaneous pregnancy included risk of late diagnosis of pregnancy (particularly in the context of anovulatory or lactational amenorrhoea), relative inability to optimise preconception health and significant logistical challenges.

After a subsequent spontaneous pregnancy, thirteen women changed their perception of their own fertility, and in a couple of cases (P1, P5) even became concerned that they were now “super-fertile”. A radical change in outlook with respect to contraception following a spontaneous pregnancy was more likely in women experiencing a very short inter-pregnancy interval. However, eight women held persistent beliefs that they had low fertility and that their spontaneous pregnancy had been a highly unlikely “fluke” event. Two of these women had a further spontaneous conception which then did change their perception of their fertility. Perceptions of high fertility across the sample were then subject to decrease with age and in response to other relevant life events e.g. iatrogenic menopause, salpingectomy etc. After spontaneous pregnancy (or pregnancies), most women in the sample (13/21) felt their families were complete, with five having more mixed or wistful feelings regarding future pregnancies and three currently pregnant.

Half of the non-pregnant women in the sample (9/18) were using barrier methods (6/18), ineffective methods (2/18) or no method at all (1/18). Three women were using oral hormonal contraception and four were using long acting reversible contraception (LARC) methods. Of the thirteen who had completed their families, seven continued to rely on barrier, ineffective or no methods of contraception (P1, P2, P7, P11, P12, P13, P14). This included two women who experienced radical changes in their perceptions of fertility (P1, P11) after spontaneous pregnancy and had therefore started to use barrier contraception after a 5-year period of no contraception use. Two women had had bilateral salpingectomies, one of whom also had an intrauterine system (IUS) for gynaecological indications and partner vasectomy. 14 women demonstrated some ongoing unmet contraceptive need, including five women dissatisfied with their current method, others who were using methods less safe or less reliable with advancing age and perimenopause and those who needed contraceptive counselling including non-contraceptive benefits.

### The infertility identity

One of the most immediate observations was the women’s beliefs in their absolute and ongoing infertility. This section explores how this mindset is developed and reinforced via the IVF process and forms an important part of women’s identities as parents after IVF. We also consider how it informs their subsequent pregnancy planning and contraception decision-making.

#### The origin

Several women had held beliefs regarding their subfertility for many years prior to trying to conceive due to early diagnoses (including polycystic ovarian syndrome (PCOS), chlamydia, and hypothyroidism), partner medical history (testicular cancer) and family history of subfertility. Factors contributing to women’s beliefs in their persistent subfertility included duration of subfertility (i.e. time spent trying to conceive) and cumulative time spent not using contraception and not conceiving (regardless of desire to conceive at that time).

“Essentially, we’ve been having unprotected sex one way or another. Okay, he’s maybe not ejaculated every single time, but we’ve been having unprotected sex for six years and I’ve never gotten pregnant.” (P6)

#### The effect of IVF

The emotive and enduring effect of IVF, was evident during many interviews with ‘flashbulb memories’ of subfertility and fertility treatment that had included pregnancy loss, life-threatening ectopic pregnancy and postpartum haemorrhage (P9, P10, P19). Five of the women became tearful during the interview and others vividly described acute sadness, e.g.:

“…when you’re going through that IVF process, you’re thinking about a lot further in the future as well. I was thinking about being a grandparent, or other people being grandparents and they’re all at family occasions, and it’s just you and your husband; everywhere you go it’s just you and your husband, there’s no children or grandchildren. So, even when you get to 60/70s and everyone else is becoming a grandma, if you haven’t had your own children then you miss out again… “(P5)

This may have been exacerbated by not having openly talked about the subject often, recently or ever, as acknowledged by several women. Three women described the ‘loneliness’ associated with IVF (P7, P9, P16), others described ‘shame’ and perceptions of an ‘unnatural’ or ‘abnormal’ conception, one woman mentioned “outing ourselves” (P20) only after her first spontaneous pregnancy and one interviewee (P3) described lying when asked directly if her twins were conceived via IVF.

**Fig. 2:**
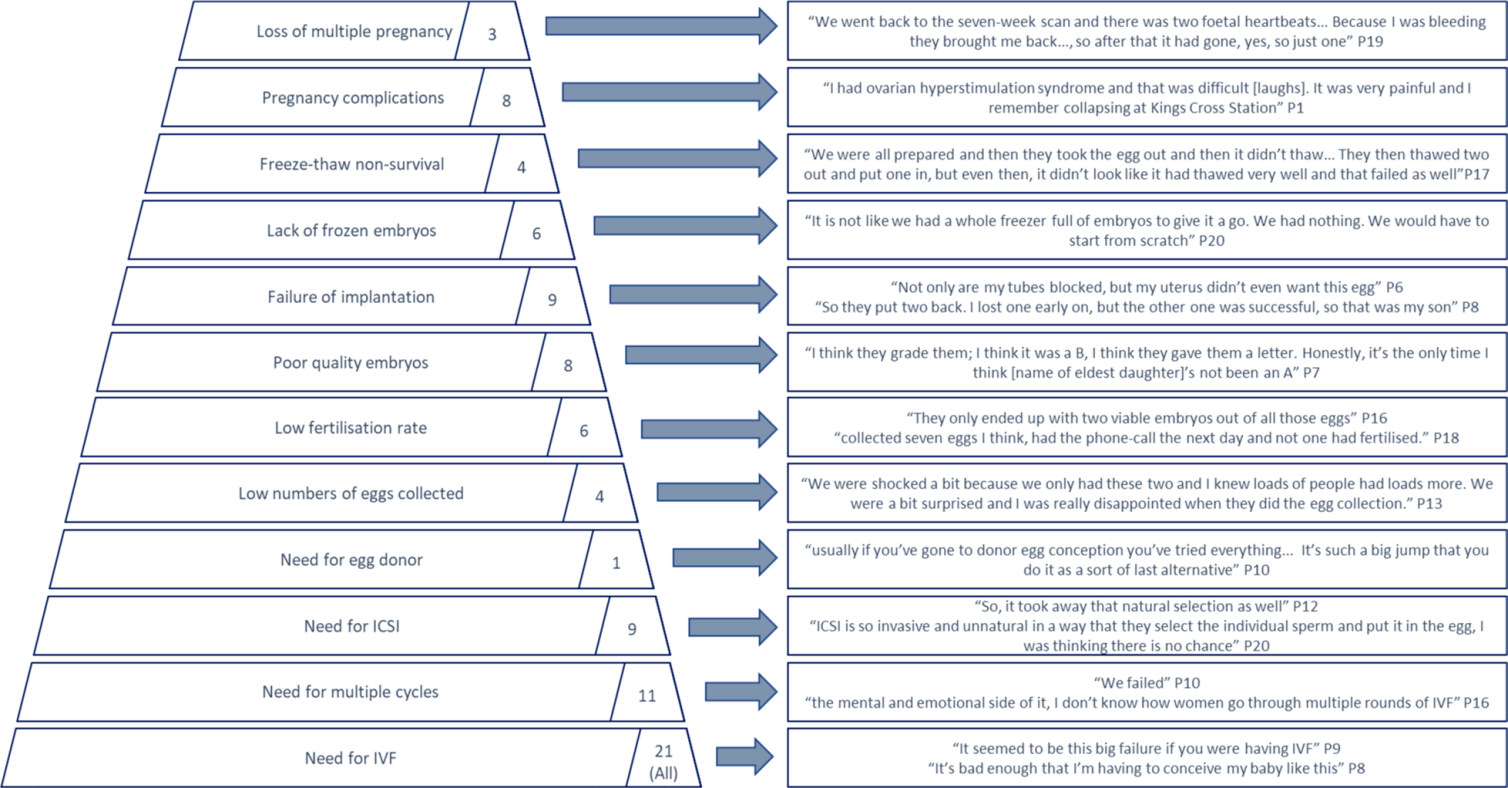
IVF "Pyramid of Failure”

Twelve specific aspects of the IVF process were identified which women associated with failure and may reinforce or confirm a woman’s belief in her persistent subfertility (Fig. 2). These include numbers of cycles (more or less than anticipated e.g. freeze/thaw non-survival), eggs (low or high with low subsequent fertilisation rate), embryos (fresh and frozen) and foetuses. Only two women had experienced a single aspect (i.e. the need for IVF) and half (11/21) had experienced five or more. These aspects were raised spontaneously by women therefore numbers in Fig. 2 are likely underestimates. Women in our sample commonly expressed nuanced feelings of personal failure associated with IVF despite ultimately achieving a livebirth. This was highlighted by the frequent use of pejorative language such as ‘funny’ or ‘dodgy’ sperm (P2, P12) and ‘dodgy’ or ‘dud’ eggs (P12, P13, P20).

#### Persistence

Women’s beliefs in their subfertility were firmly held and often persistent despite subsequent spontaneous pregnancy. One woman articulated this phenomenon as:

“I think once you go into assisted conception, once you’re in that world of assisted conception, I think you do develop a mentality of “I need assistance in order to conceive, I need fertility treatment.” (P11)

Most women expressed ‘shock’ and ‘disbelief’ when describing their initial positive pregnancy tests and many went on to take multiple confirmatory tests. Whilst these emotions may be common to women without a history of subfertility, the extent of disbelief amongst the sample was striking. One woman, even considered endocrine cancer to be more plausible:

“I remember sitting Googling “What other things give you a positive pregnancy test?” and it was endocrine cancer. So then I thought “The odds on me having endocrine cancer are higher than the odds of me being pregnant.” (P18)

The circumstances in which women took their tests also suggests the degree to which they were expecting a negative result, for example on an aeroplane, with alcohol and when unable to communicate with their partners. This is in stark contrast to scheduled testing within fertility treatment regimens. One woman, herself a GP, demonstrates lack of insight regarding her early pregnancy:

“I thought I’d put weight on because I’d been to Italy and I’d been eating loads of pasta because *I didn’t think there was any way I could be pregnant.* And then I was at work and then in the space of a week, two old ladies commented on my happy news, and I was absolutely fuming *because I wasn’t pregnant - or didn’t think I was pregnant.”* (P17)

Three women (P10, P17, P20) in the sample described their alarm at not knowing their approximate gestation at the time of their positive pregnancy test:

“It started a whole fear because I hadn’t had a period in so long I thought I could be anything between 20 weeks and just pregnant. It was terrifying. I thought, we haven’t had any of those special scans or blood tests, we haven’t had the nuchal test.” (P20)

“I don’t know if I’m beyond the timeframe of being able to have a termination if that’s what I want. Have we missed the window on all of this, and are we now going to be forced down a path that we really, really don’t think we can cope with?” (P10)

Eight women in the sample continued to hold beliefs in their subfertility despite spontaneous pregnancy, describing themselves as “very, very lucky” (P2) and their spontaneously conceived babies as a “miracle” (P18) or “little one-off fluke” (P3). Two women even expressed denial of their current pregnancies (P6, P21).

“[sigh] it’s taken me quite a long time; it’s taken me a long time to come to terms with the fact I’m pregnant. I feel a lot like it’s happening to someone else.” (P6)

“I still think I’m just maybe eating too much chocolate and not being able to fit into my jeans rather than having a pregnancy and maybe that’s just the IVF that’s made me feel like that because not thinking it would ever happen.” (P21)

#### The IVF Unicorn

The phenomenon of spontaneous pregnancy after IVF was described by one woman as the IVF “unicorn” that “happens to other people, that doesn’t happen to me” (P9).

Other women in the sample described the rarity and sensational nature of their reproductive journeys:

“I feel like I belong on the cover of Hello magazine with testicular cancer, IVF and then a [spontaneous] triplet pregnancy.” (P21)

“It is quite a story, it really is” (P19)

However, most women admitted that they did not know how likely it is to conceive spontaneously after giving birth via IVF. When asked directly 15/20 women said they did not know with four further emphasising they had “no idea”. Five women asked for the answer during or immediately after the interview. Some went on to qualify that it depends on the cause of subfertility or indications for IVF. When invited to estimate the percentage of women who conceived spontaneously within five years of giving birth via IVF, the majority (12 women) did not feel able to estimate and nine women gave estimates which varied widely (5–80%).

Most women also stated that they had not been given information regarding their chances of spontaneous pregnancy after IVF by their fertility treatment provider, except for P5 who was directly advised she had 1% chance of conceiving naturally. Women also universally said they had not received this information from healthcare professionals during maternity care, with only one woman mentioning a sonographer commenting “Oh yes, we hear of it all the time” (P18). This lack of information is in contrast with the amount of statistical information given to and sought by these women throughout their IVF treatment and their skills and abilities.

### Impact of spontaneous pregnancies

#### Short inter-pregnancy intervals

Most women (18/21) highlighted the constant time pressure associated with fertility treatment as a main driver in their decision-making during IVF including whether and when to initiate IVF and individual cycles, number of embryos to implant, whether to undertake specific investigations, participate in trials, opt for private treatment, change fertility clinics or even, in the case of one woman (P19), to stay at clinics at which they had had a traumatic experience with associated adverse pregnancy outcome. This race against time, was also identified by some women as a driver to try for a subsequent pregnancy earlier than desired, either to try and avoid further IVF or allow time for it. Women also initiated further assisted conception soon after delivery and, in one case, whilst still breastfeeding (P15). Other factors, specific to assisted conception pregnancies, which incentivise short interpregnancy intervals, include convenience and added privacy of undergoing IVF whilst still on maternity leave (P18). The availability of frozen embryos also provides women with the option of further rapid IVF.

However seven of the 16 women who were happy to have conceived spontaneously described wanting a longer gap, wanting more time to focus on their other children’s needs or describing exhaustion as older mothers with closely-spaced young children.

“I could have done with a bit more time just to be myself, to be in my own body by myself for a little bit longer. It was quite hard going straight from breastfeeding into another pregnancy, especially because we had gone through the IVF process” (P1)

#### Lack of interconception care

Out of nineteen women, asked about their preparation prior to their spontaneous pregnancy, twelve had taken no steps to improve their health. Women commonly volunteered that they were doing “none of it right “[P9] and drinking more alcohol as a marked comparison to their preparation prior to IVF, when living a “saintly” lifestyle [P9] and living “so much by the book for so long” [P7]. Seven women had taken some measures, but these were typically less stringent than prior to IVF (e.g. inconsistent folic acid use) or undertaken as part of a pre-IVF workup. P15 explained her folic acid use: “it felt like the one thing that I could do as I was waiting for treatment”. The impact of not preparing in the same way for their pregnancies can lead to feelings of regret or “what ifs”. For example, one woman questioned whether her short interpregnancy interval may have been connected to her younger daughter’s developmental delay. “…was it connected that I went into that second pregnancy too quickly?” (P1).

#### Unintended and unwelcome pregnancies

The impact of unintended and rapid repeat pregnancies included significant disruption to professional and personal plans. Several women identified themselves as “planners” which may exacerbate difficulties experienced:

“I’m quite an organised person and I like to know what’s what. So, to have something like that happen completely out of my control completely threw me. I was worried about going back to work telling them I was pregnant, I was worried about the effect it would have on my son because I wouldn’t be able to give him attention because then he was only little...” (P12)

Five women in our sample had mixed or negative feelings towards the pregnancy. This included four women (P6, P10, P11, P12), who considered termination of pregnancy and one who had a medical abortion (P11). These five women were also the oldest in our sample at the time of their spontaneous pregnancy (aged 39–43). Two further women mentioned that their partner’s feelings towards the spontaneous pregnancy were initially mixed (P20) or negative:

“when I got pregnant with the spontaneous pregnancy I think he was near breakdown, to be fair, he was absolutely distraught. It was actually the worst time ever because for me I was absolutely over the moon to be pregnant, but he didn’t want the baby and he was like, “I think we should be terminating this”. So it was a terrible time because it was like we were polar opposites over it and I kept just thinking this is all we ever wanted before and now, yes, now it’s just not… And I think had that pregnancy been successful, it would have caused problems in our relationship.” (P19).

### Factors influencing contraceptive use

**Fig. 3:**
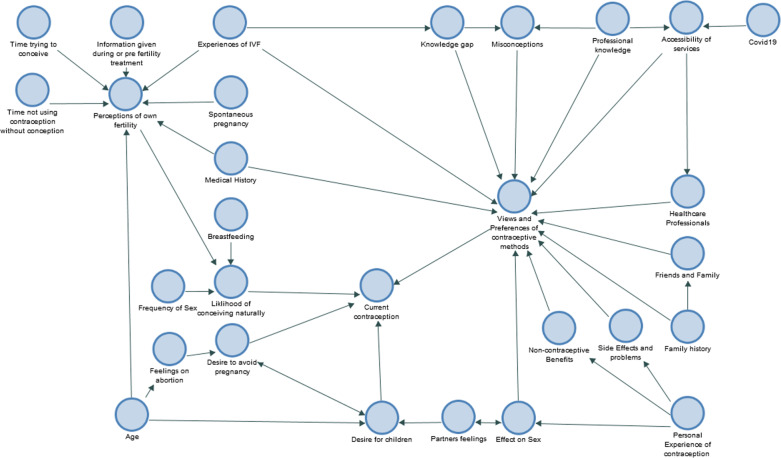
Factors influencing contraceptive choice in women having had IVF

This section explores contraception use among the women in the sample and what influenced their choices. Figure 3 shows the complex, interrelated and dynamic influences affecting a woman’s contraceptive choices after IVF. Reliance on less effective methods (condoms or withdrawal) after spontaneous pregnancy was justified as familiar, accessible, a ‘bridging’ method whilst considering other options, a ‘back-up’ method in the context of iatrogenic menopause or ‘good enough’ in the context of age-related declining fertility. However for some women consistent use of barrier methods represented a step change from non-contraception use and therefore a large relative increase in the efficacy of their contraception. The main drivers of current contraception choices were identified as women’s feelings regarding:

- 1. Desire for a child
- 2. Likelihood of natural conception
- 3. Contraceptive methods

#### Desire for a child

A woman’s desire for a child was, in turn, subject to many, internal and external influences and can be considered as a pendulum swinging between extremes of strong desire to be pregnant and avoid pregnancy with significant time spent in a position of mixed feelings. This was evidenced, when asking women their views on future pregnancy:

“Mortified, terrified, I wouldn’t keep it.” (P12)

“I would rather not get pregnant now, but if I did I would accept it. My husband would be delighted, I would probably be a bit more mixed.” (P3)

“So I would love more children, I really would, but my husband really doesn’t want any more… …And I still want more, but I’ve kind of come to the conclusion that that is the end of it.” (P19)

Influences included number of own siblings, ‘maternal’ self-identity, partners’ feelings, and trigger events e.g. family childhood illness. Number of existing children, and reproductive experiences were also strong influencing factors, with women expressing contrasting views such as relief or guilt at having one child and “greed” for wanting more. Personal medical history also played a major part in two women’s wish to avoid pregnancy:

“I would worry about it causing breast cancer again, because it’s an oestrogen effect. I’ve got two children that I’ve got to stay alive for, so that’s my aim.” (P12)

“I also had a big bipolar relapse last year and I would be quite worried about that, coming off lithium again. I would be really not very happy and quite frightened.” (P4)

However, ultimately age appears a universally dominant influence with women increasingly citing this factor as their wish to avoid pregnancy. Paternal age was also mentioned by women highlighting that they are considering not just pregnancy risk but the longer-term implications of raising children. Two women described openness to termination of pregnancy as part of their decision-making logic to accept a less effective method. The personal stance on abortion by women in the sample was also influenced by age.

#### Likelihood of natural conception

When considering contraceptive non-use, perception of own fertility is consistently the most influential factor. Frequency of sex and breastfeeding status also affected views on likelihood of natural conception and subsequent contraceptive choices.

#### Views on contraceptive methods

The views and preferences regarding specific contraceptive methods were informed by:

i. personal and professional experience of contraceptive methods
ii. contraceptive experience of friends and family
iii. information given by healthcare professionals and other sources

Personal experience of contraception was seen to have a strong, long-lasting influence on current contraceptive choices with 17/21 women mentioning side effects, even decades previously, and therefore choosing to avoid the method long-term as a result. Previously experienced non-contraceptive benefits also influenced contraception preferences but were more subject to change over time (e.g. women developing medical contraindications or new side effects) and so women were not always able to resume these methods satisfactorily. The impact on sex and partner’s preferences also influenced choice of method.

Contrasting examples demonstrated the influence of a woman’s experiences as a fertility patient on views of contraceptive methods:

“I know you can have an implant and coil and things like that. I’m not a massive fan; those sorts of things feel a bit invasive to me, and maybe because of having had fertility treatment I’m a bit averse to things like that.” (P11)

“I figure that if you have gone through fertility treatment that you could certainly cope with a coil insertion.” (P15)

Misconceptions amongst our sample most commonly related to the effectiveness of breastfeeding as contraception:

“he was down to maybe one or two feeds a day, and I guess that was my safety net gone then, but I didn’t really realise it.” (P11)

Professional knowledge influenced contraceptive preferences:

“It’s interesting, because I am Family Planning Lead in my (GP) practice, so I fit coils and implants. I would never have a coil [laughs]. Oh, the thought; it gives me shivers. So, I would never have an IUD [Intrauterine device] … …when you’re a Medic and you have that – not necessarily better knowledge – but a different skew on things, don’t you?” (P17)

Family history of breast cancer was cited by two women as reason to avoid hormonal methods. Experience of family and friends generally was cited frequently as a reason for contraceptive preferences. This influence persisted despite insight expressed as to the limitations of anecdotal or outdated accounts. Information given by healthcare professionals also influenced contraceptive decisions, although these were challenged and revisited over time.

During fertility treatment, none of the women reported receiving any information on contraception. Similarly, none of the women in the sample reported any contraception counselling during antenatal care. This included the three women who were pregnant at the time of interview. No women reported any postnatal contraception counselling, except for routine, “tick-box” questions as described by ten women or platitudes such as “take care” or “watch out”. Four women said it had been raised as “a joke” sarcastically, colluding with views of persistent subfertility.

“they were like, ‘It is not a problem for you, haha, but we do have to tell you this and sign it off.’ There was no indication whatsoever and people were quite unhelpful about it in a jovial way, but it can hurt. It was mentioned because it was a tick boxing exercise, not because they genuinely were giving me the impression that we could ever get pregnant naturally.” (P20)

Women also commonly described being given “a leaflet” as part of routine procedure and in place of a discussion, with one adding “I probably put it in the recycling” (P11). Others felt this was irrelevant “I think I was given a leaflet. And I just remember thinking, right, that doesn’t mean anything to me, it’s an IVF baby” (P19).

Only a handful of women recalled it being discussed at the GP six-week check, with competing priorities at that time. Three of the five women feeling that this was insistent or unwelcome and two women describing this limited to an interrogatory “what are you going to do about contraception?” No women reported helpful discussions at that time. The woman in our sample who had had a termination of spontaneous pregnancy after IVF pregnancies reported no follow up on contraception post termination (P11).

### Women’s reflections and recommendations

In this final theme, we present the women’s views of how, when and by whom postnatal contraception counselling should be delivered.

#### Likelihood of spontaneous conception

Women consistently identified a need for their IVF journey to be explicitly acknowledged by healthcare professionals, and to be informed that subsequent spontaneous pregnancy *is* possible. They unanimously agreed that evidence-based, statistical information as to how common this phenomenon is would have been helpful in their subsequent family planning decision-making.

Most women identified the need for information to be tailored to cause of subfertility, since, as one woman stated “by the time they’ve been through IVF, most women understand that they’re not all the same. Your likelihood of success depends on several factors, doesn’t it, so that your fertility afterwards is also going to depend on those factors” (P8). The need to frame this information, in terms of avoiding unplanned pregnancy as opposed to chances of success without treatment, was also raised by two women to avoid giving ‘false hope’.

#### The role of fertility treatment providers

There was also consensus that contraception counselling was inappropriate during fertility treatment:

“I just wanted a baby, didn’t dare to think that we might be able to have more than one baby, and I think it would have upset me for somebody to start talking to me about next time, until I’d had the baby.” (P8)

However, two women (P1, P14) mentioned that information on the likelihood of natural conception after IVF would have been useful during fertility treatment and several women (7/21) asserted that fertility providers could and should do more after delivery. One woman, herself a GP, asserts that information (both regarding risks of conception and contraception) needs to come from the fertility team as “more likely to have weight” (P15). Four women (P6, P15, P20, P21) identified a good time for giving this information as concurrent with the post pregnancy outcome questionnaire, part of clinic’s compulsory HFEA reporting requirements.

#### Antenatal contraception counselling

When considering antenatal contraceptive counselling by maternity services, there were again wide-ranging views. Competing medical priorities and concerns, for example, high-risk twin pregnancies were highlighted, and some felt strongly that they would not have been receptive to contraceptive counselling at any point during pregnancy:

“I don’t think that when I was pregnant with my IVF pregnancy, if someone had talked to me at that point about contraception, it wouldn’t have been well received. It’s fine afterwards when you’re sitting there with a baby in your arms, it’s not okay when you’re still wondering if you’re going to go home with a baby. You’re so anxious that it might not work out, even when you’re 36, 37 weeks, I wouldn’t have received that well.” (P8)

Two women supported antenatal contraception information (P12, P14), both qualifying this as “well into the pregnancy” or “on discharge from fertility services”. The suggestion that antenatal contraceptive counselling after IVF is better received at later gestations may reflect the more gradual process of belief in their ongoing pregnancy, described by several women:

“I didn’t really believe it until right towards the end. I was like, this cannot happen. As you progress through the pregnancy, you believe it more and more.” (P20)

“Every step of the way, I thought something was going to go wrong, so I was a bit worried about it, I suppose, yes. I freaked the bloke out in Mothercare, because you have to buy stuff before the baby arrives otherwise you’d need… So, I said “Well, what if we don’t need it?” and he looked at me with a blank face and I said “Well, what if the baby dies before it’s born? What do I do with the…?” because it’s a lot of money.” (P12)

#### Postnatal contraception counselling

Within the immediate and early postnatal period there was again a spread of views. One woman (P21) acknowledged a need for contraception counselling on the postnatal ward in contrast to multiple views that contraception seemed ludicrous or irrelevant in that setting, together with a desire to finally stop thinking about fertility and enjoy parenthood. Several women suggested a period longer than and specifically outside of the six-week GP check (six weeks -six months). Only two women (P4, P8), both GPs, recommended the current, six-week check as the right time. However two women (P1, P13) specified that this should occur before weaning and several women also raised the importance of information relating to the role and limitations of breastfeeding as contraception. Women also expressed a desire for a dedicated conversation about contraception:

“Rather than as an add-on conversation to a quick “How are you doing? How are you feeling?” Checking you’re not leaping out windows or anything due to lack of sleep. I think maybe if it had been a separate and isolated conversation… (P7)

## Discussion

The most striking finding from our study was the widespread perception of low fertility across the sample and how firmly and persistently these beliefs were held despite subsequent, spontaneous pregnancy. While the ‘success’ of IVF is often defined in terms of livebirth [14], we identified aspects of the IVF process, which women associated with failure *despite* livebirth. These may reinforce or confirm a woman’s belief in her ongoing subfertility. Fixed belief in subfertility was a major barrier to contraception use resulting in rapid repeat, unintended and unwelcomed pregnancies. After spontaneous pregnancy, despite desire to avoid pregnancy and any changes in perception of fertility, the use of contraception and the most effective (long-acting, reversible) methods remained low.

A recent, small qualitative study, exploring the effects of infertility and IVF on couples’ experiences of early parenthood, similarly found the experience of successful IVF to have a significant and long-lasting effect on couples. This study of sixteen UK heterosexual, non-donor partnerships described how this experience shaped and challenged parental identity and was a source of anxiety and particular difficulty when considering a sibling for their child [10]. The prolonged and pervasive impact on the identity of those parenting after infertility is also asserted in earlier studies [11, 12]. To our knowledge, this is the first study of views and experiences of contraception among women having successful IVF.

It is important to recognise that unplanned, spontaneous pregnancies after IVF are not universally welcomed and that the ability to plan and space pregnancies is as important to women who have had IVF as those who have not. However, the high proportion of short interpregnancy intervals after IVF birth demonstrates a lack of effective postnatal contraception use in this group. Experience as a fertility patient may contribute to a knowledge gap in contraception, given extended periods of time without personal use and relative inexperience with specific or newer methods. They may also be less aware of sources of information and less likely to have discussed this with peers or healthcare professionals. Similarly, it is possible that women after IVF births may be less aware of the recommended interpregnancy interval [13], however, there is evidence to suggest that there are low levels of knowledge of postnatal contraception among postnatal women in the UK generally [14].

The lack of information on the probability of spontaneous pregnancy and contraception received by the women after IVF birth is certainly in contrast with the amount of statistical information given to and sought by women throughout their IVF treatment. This suggests that the major challenge to provision of contraceptive care is their own perception of pregnancy risk. For a woman who thinks she cannot conceive naturally, not pursuing assisted conception can be considered an active contraceptive choice. Without first acknowledging a woman’s IVF experience and informing them of the chances of spontaneous conception and reasons for avoiding rapid, repeat pregnancy, they are unlikely to engage with contraceptive counselling. The views expressed by women in our sample that fertility providers could and should do more after delivery mirrors sentiments expressed in recent online media in which women felt generally "cut adrift" after IVF and call on fertility clinics to extend the support they provide [15]. GPs, community midwives, health visitors, maternity staff and termination services also undoubtedly have a part to play. However a consistent message is that these healthcare professionals must all be armed with knowledge specific to women having had IVF, in order to recognise and meet their needs.

Our sample demographics were seen to broadly reflect that of women having IVF in the UK. The average age of UK IVF patients has remained stable over the past two decades, at 34 years [16] and the ethnicity of women having IVF in the UK in 2018 identified as 66% White, 19% Asian, Black, Mixed or Other, with the remaining 15% not stated [17]. There is also UK evidence that women of higher education status are more likely to report fertility problems [18]. With good geographical sample spread across the UK, it is likely that the findings and recommendations are generalizable nationwide. Having interviewed women at a single point in time, we are relying on their reflection of past events, introducing potential recall bias. However our sample included women who were currently pregnant as well as women further towards the end of their reproductive life to capture a range of views and experiences over time. Moreover, since the focus of this study was the contraceptive need of women *after* IVF, only salient information regarding their fertility journeys which impacted their subsequent contraceptive decisions was required. Reflecting the recruitment strategy, our sample contained a high proportion of health professionals who may have better knowledge of contraception than other women having IVF. However such knowledge was not considered a valid exclusion criterion, relevant professional experience was asked about directly where appropriate in the interviews and provided further insights. Moreover, some medical doctors in the sample felt they had outdated knowledge or skewed views towards contraception. Finally, it is possible that a woman’s perceived need for contraception may have been altered by invitation to this study, indeed this was acknowledged explicitly by one woman (P13) who said it had prompted her to book a contraception consultation. However this was felt unlikely to have changed their prior views regarding contraception.

The interviewer (AT) was a white British woman in her early 40s, mother of two children and an academic clinical doctor in the specialism of sexual and reproductive health. AT’s professional background was communicated to potential participants prior to the interview with a link to a brief biography contained in the study participant information sheet. The personal characteristics of the interviewer mirrored many of those of the participants which was felt to be helpful in facilitating sharing of personal and emotive topics, including some not widely shared within their partnerships. It was however noted that some responses around contractive choices were couched apologetically:

“…the coil just doesn’t sound very comfortable. I don’t really like the sound of it, sorry [laughs].” (P2)

“This is where I feel I will get chucked out of the contraception club” (P14)

This may have been due to the perception that a doctor specialising in contraception may judge these views but, given the fact that women reported low levels of contraception use throughout the interviews, it probably did not materially alter the content of their responses.

## Conclusions

The contraceptive needs of women having IVF pregnancies are real but are being overlooked. The consequences of this oversight include unwelcome and rapid repeat pregnancies, while the impacts on women and their families are wide-ranging and profound. Fertility services should take responsibility for providing information on the likelihood of subsequent spontaneous pregnancy and the need to consider postnatal contraception. They are well placed yet not incentivised to do so. Potential barriers to contraception counselling and provision that are specific to women after IVF include firm and persistent beliefs in subfertility, lack of knowledge of likelihood of spontaneous pregnancy and inherent drivers towards shorter inter-pregnancy intervals. Maternity and community healthcare professionals must first address these issues in order to support women in meeting their contraceptive needs.

## Data Availability

The data that support the findings of this study are not publicly available in order to protect the privacy of the individual participants. This is due to the sensitive subject matter and potentially identifiable reproductive experiences. Anonymised data are however available from the corresponding author upon reasonable request.

## Abbreviations

ART: Assisted reproduction technology
DSH: Data safe haven
GP: General practitioner
HFEA: Human fertilisation and embryology authority
IUD: Intrauterine device
IUS: Intrauterine system
IVF: In vitro fertilisation
LARC: Long acting reversible contraception
NHS: National Health Service
PCOS: Polycystic ovarian syndrome
PGD: Preimplantation genetic diagnosis
TOP: Termination of pregnancy
UCL: University College London

## References

[1] Human Fertilisation and Embryology Authority. Fertility treatment 2018: trends and figures. 2020.

[2] More than 8 million babies born from IVF since the world's first in 1978 [press release]. ScienceDaily: European Society of Human Reproduction and EmbryologyJuly 3, 2018.

[3] Richardson A, Taylor M, Teoh J, Karasu T (2020). Antenatal management of singleton pregnancies conceived using assisted reproductive technology. Obstet Gynaecol.

[4] Fertility problems: assessment and treatment. National Institute for Health and Care Excellence; 2013 (last updated 2017). 32134604

[5] ElMokhallalati Y, van Eekelen R, Bhattacharya S, McLernon DJ (2019). Treatment-independent live birth after in-vitro fertilisation: a retrospective cohort study of 2,133 women. Hum Reprod.

[6] Canning K. The Surprising Reason Why Some Women Get Pregnant Naturally After Undergoing IVF or Adopting. Health.com2021.

[7] Anonymous. https://www.telegraph.co.uk/women/life/spent-years-trying-conceive-had-abortion/. Stella Magazine. 2020. accessed 30 Aug 2020

[8] Ritchie J, Spencer L (1994). Qualitative data analysis for applied policy research. Analysing qualitative data.

[9] Boivin J, Harrison C, Mathur R, Burns G, Pericleous-Smith A, Gameiro S (2020). Patient experiences of fertility clinic closure during the COVID-19 pandemic: appraisals, coping and emotions. Hum Reprod.

[10] Allan H, Mounce G, Culley L, van den Akker O, Hudson R (2021). Transition to parenthood after successful non-donor in vitro fertilisation: The effects of infertility and in vitro fertilisation on previously infertile couples' experiences of early parenthood. Health (London, England: 1997)..

[11] Olshansky E (2003). A theoretical explanation for previously infertile mothers' vulnerability to depression. J Nurs Scholarship.

[12] Sandelowski M (1995). A theory of the transition to parenthood of infertile couples. Res Nurs Health.

[13] World Health Organization (2013). Programming strategies for postpartum family planning.

[14] Thwaites A, Logan L, Nardone A, Mann S. Immediate postnatal contraception: what women know and think. BMJ Sex Reprod Health. 2018. 10.1136/bmjsrh-2018-200078 30463845

[15] Moss R. This Is How It Feels To Be 'Cut Adrift' After IVF, Say Women. HuffPost UK. 2020 Nov 2; 2020.

[16] Omer N, Hill E. From the first IVF baby to now – how the fertility industry grew. Inside ‘Big Egg’. Tortoise Media; 2020.

[17] New figures show low uptake of fertility treatment among BAME communities [press release]. Human Fertilisation and Embryology Authority; 2019.

[18] Morris M, Oakley L, Maconochie N, Doyle P (2011). An investigation of social inequalities in help-seeking and use of health services for fertility problems in a population-based sample of UK women. Hum Fertil.

